# Prevalence and Associated Risk Factors of Neonatal Sepsis in a Neonatal Intensive Care Unit of a Tertiary Hospital in Bangladesh: A Cross‐Sectional Study

**DOI:** 10.1002/hsr2.72661

**Published:** 2026-06-10

**Authors:** Fahmida Akter, Halima Khatun Munni, Md. Abdur Rahman Ripon, Sujan Banik, Mohammad Salim Hossain

**Affiliations:** ^1^ Department of Pharmacy Noakhali Science and Technology University Noakhali Bangladesh

**Keywords:** Bangladesh, neonatal sepsis, prevalence, risk factors, tertiary hospital

## Abstract

**Background and Aims:**

Neonatal sepsis is a leading cause of neonatal morbidity and mortality in low‐ and middle‐income countries, including Bangladesh. This study aimed to determine the prevalence and identify associated risk factors of neonatal sepsis among neonates admitted to a tertiary hospital in Bangladesh.

**Methods:**

A hospital‐based cross‐sectional study was carried out at the Institute of Child and Mother Health (ICMH), Dhaka, Bangladesh, from December 2023 to July 2024. A total of 125 neonates admitted to the neonatal intensive care unit (NICU) were included in this study through systematic random sampling. Data on sociodemographic, maternal history, and neonatal clinical characteristics were collected using a structured questionnaire.

**Results:**

Neonatal sepsis was diagnosed in 69.6% (*n* = 87) of the neonates, with early‐onset sepsis accounting for 60.9% and late‐onset for 39.1%. Key risk factors significantly associated with neonatal sepsis included neonates with low birth weight (1.5−2.4 kg) (OR: 6.86, 95%CI: 2.22−21.2, *p* = 0.000), lower maternal education (OR: 13.6, 95%CI: 2.27−81.3, *p* = 0.002), low family income (OR: 2.71, 95%CI: 1.20−6.10, *p* = 0.025), and home delivery (OR: 3.16, 95%CI: 1.39−7.18, *p* = 0.009).

**Conclusions:**

This study reveals a high prevalence of newborn sepsis in a tertiary care setting in Bangladesh, which highlights the urgent need for preventive strategies. Strengthening maternal education, promoting institutional deliveries, and improving socioeconomic conditions are crucial for reducing neonatal sepsis. Further large‐scale multicenter studies are needed to assess the prevalence of neonatal sepsis in Bangladesh.

## 1 Introduction

Neonatal sepsis is a life‐threatening clinical syndrome characterized by systemic signs of infection and the presence of bacterial or fungal pathogens in the bloodstream, occurring in the first month of life [1]. Globally, it is still a major public health challenge, despite the modern advances in neonatal care, particularly in low‐ and middle‐income countries (LMICs) like Bangladesh, where healthcare infrastructure faces challenges in addressing maternal and neonatal health [2]. According to the World Health Organization (WHO) report, each year around 4 million children die during the first 4 weeks of their lives, and the rate of neonatal mortality in LMICs is 20 per 1000 live births compared to 3 per 1000 in high‐income countries [3]. It is considered the leading cause of neonatal mortality, accounting for an estimated 30%–50% of neonatal deaths in LMICs each year [4, 5].

The global prevalence of neonatal sepsis is highest in South Asia and sub‐Saharan Africa [6]. Recent systematic reviews and global analyses have highlighted that severe neonatal infections continue to contribute significantly to neonatal morbidity and mortality, with substantially higher incidence rates in LMICs compared to high‐income countries [7]. In 2013, South Asia was responsible for 38.9% of the total number of deaths that were attributed to sepsis in neonates [8]. There are two types of neonatal sepsis: early‐onset neonatal sepsis (EONS), occurring within the first 72 h of birth, and late‐onset neonatal sepsis (LONS), occurring after the first 72 h. It is believed that germs transmitted from mothers are responsible for EONS, while LONS is attributed to horizontal transmission from the environment, caregivers, or both. It has been reported that around 62% of infections in South Asia occur within 72 h of life, with an incidence of 9.8 per 1000 live births [6]. This is 10 times higher than the incidence of LONS reported in a large nationwide study conducted in the United States [9].

Bangladesh, a developing country in South Asia, has significantly reduced the trend of neonatal mortality over the past decades, with rates declining from 50.2 to 31.9 per 1000 live births between 2000 and 2017 [10, 11]. Despite this progress, neonatal mortality in Bangladesh remains significantly higher than in developed countries, with neonatal sepsis being a critical contributor [8]. A previous study reported that the prevalence of sepsis among neonates admitted to public hospitals in Bangladesh was 69.35% [12]. Risk factors associated with neonatal sepsis reported in the region include preterm birth, low birth weight, prolonged rupture of membranes, perinatal asphyxia, and poor maternal healthcare during pregnancy and delivery [13]. While previous studies have reported potential risk factors for neonatal sepsis, there are still knowledge gaps regarding region‐specific up‐to‐date data that reflect the current socio‐demographic status and improved healthcare settings in Bangladesh. To address these gaps and to up‐to‐date scenario, this cross‐sectional study aims to investigate the prevalence and associated risk factors of neonatal sepsis in the neonatal intensive care unit (NICU) of a selected tertiary hospital in Bangladesh. This study hypothesizes that neonatal sepsis remains highly prevalent in Bangladesh, and low maternal education, home delivery, and low birth weight would be the strongest risk factors for neonatal sepsis in this setting.

## 2 Methods and Materials

### 2.1 Study Design, Setting, and Period

This cross‐sectional study was conducted at the Institute of Child and Mother Health (ICMH), located in Matuail, Dhaka, Bangladesh. ICMH is a major national‐level institute dedicated to the welfare of children and mothers in Bangladesh under the Ministry of Health and Family Welfare, to provide high‐quality care for critically ill neonates. The hospital operates a specialized Level III NICU that delivers advanced neonatal care in Bangladesh, with over 90 beds allocated to pediatric and neonatal special care units. ICHM in Dhaka manages a large number of neonatal admissions each year, making it the busiest neonatal service in the nation. During the study period from December 2023 to July 2024, a total of approximately 420 neonates were admitted to the NICU. This study was carried out over a period of 7 months.

### 2.2 Study Definitions

Neonatal sepsis is the systemic inflammatory response syndrome with infection and is identified based on clinical and laboratory criteria. Suspected sepsis was confirmed based on the presence of at least one of the following clinical features: fever, hypothermia, poor feeding, lethargy, hypotonia, brady/tachycardia, or respiratory distress. Confirmed sepsis was diagnosed based on positive blood culture results or other laboratory markers, such as elevated C‐reactive protein, procalcitonin levels, or total leukocyte count abnormalities.

In this study, neonatal sepsis cases were classified into culture‐positive (confirmed) sepsis and culture‐negative (clinical) sepsis. Culture‐positive sepsis was defined as neonates with compatible clinical symptoms and a positive blood culture result. Culture‐negative sepsis refers to neonates who presented with clinical signs of infection and abnormal laboratory markers suggestive of infection but had negative blood culture results. Both categories were included in the estimation of neonatal sepsis prevalence.

### 2.3 Study Sample, Size, and Sampling Technique

This study considered all neonates as study samples who were admitted to the NICU of the selected hospital during the study period from December 2023 to July 2024. The sample size was calculated using the following single population proportion formula, considering a previously reported neonatal sepsis prevalence of 69.35% in Bangladesh [12], with a 95% confidence interval and an 8% margin of error. The calculated minimum sample size was 122. A systematic random sampling technique was employed in this research to select the study participants. Initially, one neonate was selected randomly from the admitted neonates, and then every third neonate was included in the study until the required sample size was achieved. In total, 125 neonates were included as study subjects during the study period.

$$n=\frac{Z^{2}.\;p.\;(1-p)}{d^{2}}$$

### 2.4 Data Collection

A structured questionnaire was prepared for data collection based on previously published literature and medical record reviews. The questionnaire consisted of three sections: sociodemographic characteristics, maternal information, and neonatal clinical features. Maternal data included age, education, occupation, family income, and delivery‐related information (e.g., mode of delivery and place of delivery). Household economic status was categorized based on monthly family income, where families with an income of ≤ 25,000 Bangladeshi Taka (BDT) were classified as low‐income, and those with an income of > 25,000 BDT were categorized as higher‐income households. Neonatal data included age, sex, birth weight, gestational age, residence, and clinical signs of sepsis. Birth weight was categorized according to standard neonatal classification as extremely low birth weight (ELBW, < 1.0 kg), very low birth weight (VLBW, 1.0–1.4 kg), low birth weight (LBW, 1.5–2.4 kg), and normal birth weight ( ≥ 2.5 kg) [14].

### 2.5 Data Analysis

All collected data were coded, entered, and analyzed using Statistical Package for the Social Sciences (SPSS) software version 23. Descriptive statistics were employed to summarize the demographic and clinical characteristics of the study participants. In this study, categorical variables were presented as frequencies and percentages. The prevalence of neonatal sepsis was calculated as the proportion of neonates diagnosed with sepsis among the total number of neonates admitted during the study period, and was expressed as a percentage. Bivariate analysis was performed using the *χ* ^2^ test to assess the association between neonatal sepsis and potential risk factors. The strength of association was evaluated based on observed distribution differences across categories. A *p* value of less than 0.05 was considered statistically significant.

### 2.6 Ethical Considerations

Ethical approval was obtained from the respective ethical review committee of the Noakhali Science and Technology University (NSTU) following the submission of the study protocol. Parental informed consent was obtained prior to the inclusion of neonates in the study. Parents have the full right to withdraw their child from the research activity at any time. This study also maintained the privacy of the participants during data collection. The study adhered to the principles outlined in the Declaration of Helsinki.

## 3 Results

### 3.1 Characteristics of the Neonates

Table 1 presents the demographic and clinical characteristics of the 125 neonates involved in the study. The majority of the neonates (77.6%, *n* = 97) among the respondents were between 0 and 7 days old, while the remaining 22.4% (*n* = 28) were between 8 and 28 days old. The male and female percentages were 56% (*n* = 70) and 44% (*n* = 55), respectively. Slightly more than half of the neonates (52%, *n* = 65) resided in rural areas, while 48% (*n* = 60) were from urban areas, showing a fairly balanced distribution between rural and urban populations. Neonates' birth weight data indicated that 19.2% (*n* = 24) of the neonates had very low birth weight (1.0–1.4 kg), 42.4% (*n* = 53) had low birth weight (1.5–2.4 kg), and 38.4% (*n* = 48) had a normal birth weight ( ≥ 2.5 kg). No cases of extremely low birth weight (< 1.0 kg) were observed in the study population. The majority of the neonates (72%, *n* = 90) were born at term, while 28% (*n* = 35) were preterm, which is notably higher than the national average and reflects the high‐risk nature of NICU admissions.

**Table 1:** Characteristics of the study subjects (*N* = 125).

Variables	Categories	Frequency (*n*)	Percentage (%)
Age (days)	0 to 7 days	97	77.6
	8 to 28 days	28	22.4
Sex	Male	70	56
	Female	55	44
Residence	Rural	65	52
	Urban	60	48
Birth weight (kg)	1.0–1.4	24	19.2
	1.5–2.4	53	42.4
	≥ 2.5	48	38.4
Gestation age	Preterm	35	28
	Term	90	72

### 3.2 Characteristics of the Mothers

Table 2 represents the sociodemographic characteristics of the mothers of the neonates. The majority of the mothers (72%, *n* = 90) were aged between 20 and 30 years, while 24% (*n* = 30) were under 20 years, and only 4% were above 30 years. Nearly half of the mothers (46.4%, *n* = 58) had completed primary education, whereas 39.2% (*n* = 49) had a level of secondary education, and 8.8% were illiterate. Most mothers (81.6%, *n* = 102) were housewives, and 70.4% (*n* = 88) belonged to low‐income families according to the predefined household income classification. In terms of delivery mode, 77.6% (*n* = 97) had normal deliveries, while 22.4% (*n* = 28) underwent cesarean sections (C‐sections). Forty‐eight percent (*n* = 60) of the deliveries occurred at home, whereas 52% (*n* = 65) took place in hospitals.

**Table 2:** Sociodemographic characteristics of the mothers (*N* = 125).

Variables	Categories	Frequency (*n*)	Percentage (%)
Maternal age (years)	< 20 years	30	24
	20–30 years	90	72
	> 30 years	5	4
Education	Illiterate	11	8.8
	Primary level	58	46.4
	Secondary level	49	39.2
	Above secondary	7	5.6
Occupation	Housewife	102	81.6
	Employed	23	18.4
Family income	Low	88	70.4
	High	37	29.6
Mode of delivery	Normal	97	77.6
	C‐section	28	22.4
Place of delivery	Home	60	48
	Hospital	65	52

### 3.3 Prevalence of Sepsis

**Figure 1:**
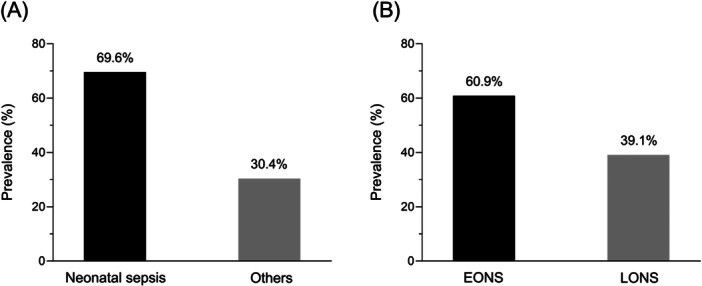
Prevalence of neonatal sepsis (A) and their distribution according to neonatal sepsis types (B) among the study subjects.

Among 125 neonates who were included in the study, 87 (69.6%) were diagnosed with neonatal sepsis, while 38 (30.4%) were afflicted with other diseases, including meningitis, perinatal asphyxia, neonatal jaundice, respiratory distress, and transient tachypnoea (Figure 1A). As shown in Figure 1B, a clear distinction was observed between the types of neonatal sepsis of EONS and LONS. Among the 87 neonates who were affected by sepsis, EONS accounted for 53 cases (60.9%), while LONS was observed in 34 cases (39.1%).

### 3.4 Factors Associated With Neonatal Sepsis

The study identified several significant risk factors that were strongly associated with neonatal sepsis in this study population (Table 3). Neonates' birth weight showed a strong association with sepsis (*χ* ^2^ = 19.4, *p* = 0.000). Neonates with low birth weight (1.5–2.4 kg) had a substantially higher risk of sepsis (OR = 7.46, 95% CI: 2.52–22.1) compared to those with normal birth weight (at least 2.5 kg). Maternal education had a significant association with neonatal sepsis (*χ* ^2^ = 14.3, *p* = 0.002), as neonates born to mothers with only primary education were significantly more likely to develop sepsis than those whose mothers had education above secondary level (OR = 13.6, 95% CI: 2.27–81.3). In addition, neonates from low‐income families had an increased risk of sepsis than those from higher‐income families (OR = 2.71, 95% CI: 1.20–6.10, *χ* ^2^ = 5.00, *p* = 0.025). On the other hand, the prevalence of sepsis was significantly threefold higher in neonates born at home than those born in hospitals (OR = 3.16, 95% CI: 1.39–7.18, *χ* ^2^ = 6.88, *p* = 0.009). However, other factors, such as neonatal age, gender, residence, gestation age, maternal age, maternal occupation, and mode of delivery, exhibited no significant associations with neonatal sepsis. These findings highlight the influence of socioeconomic and healthcare access factors on neonatal outcomes.

**Table 3:** Factors associated with neonatal sepsis in the study.

Variables	Categories	Total (*N*)	Sepsis *n* (%)	Odd ratio (95% CI)	*χ* ^2^	*p* value
Age (days)	0–7	97	64 (65.9)	0.97 (0.38–2.47)	2.68	0.309
	8–28	28	23 (82.1)			
Sex	Male	70	52 (74.3)	1.64 (0.71–3.82)	1.19	0.276
	Female	55	35 (63.6)			
Residence	Rural	65	47 (72.3)	1.30 (0.60–2.80)	0.240	0.623
	Urban	60	40 (66.7)			
Birth weight (kg)	1.0–1.4	24	12 (50.0)	0.78 (0.29–2.08)	19.4	0.000
	1.5–2.4	53	48 (90.6)	7.46 (2.52–22.1)		
	≥ 2.5	48	27 (56.3)	1		
Gestation age	Preterm	35	21 (60.0)	0.54 (0.23–1.24)	1.53	0.215
	Term	90	66 (73.3)			
Maternal age (years)	< 20 years	30	23 (76.7)	1.64 (0.63–4.26)	1.33	0.514
	20‐30 years	90	60 (66.7)	1		
	> 30 years	5	4 (80.0)	2.00 (0.21–18.7)		
Education status	Illiterate	11	7 (63.6)	4.37 (0.56–33.9)	14.3	0.002
	Primary level	58	49 (84.5)	13.6 (2.27–81.3)		
	Secondary level	49	29 (59.2)	3.63 (0.63–20.6)		
	Above secondary	7	2 (28.6)	1		
Occupation	Employed	23	17 (73.9)	1.29 (0.47–3.59)	0.061	0.805
	Housewife	102	70 (68.6)			
Family income	Low	88	67 (76.1)	2.71 (1.20–6.10)	5.00	0.025
	High	37	20 (54.1)			
Mode of delivery	Normal	97	71 (73.2)	2.04 (0.77–5.32)	1.94	0.163
	C‐section	28	16 (57.1)			
Place of delivery	Home	60	49 (81.7)	3.16 (1.39–7.18)	6.88	0.009
	Hospital	65	38 (58.5)			

## 4 Discussion

This study illustrates the prevalence of neonatal sepsis in Bangladesh, particularly in a tertiary care setting, and emphasizes the critical role of maternal, neonatal, and sociodemographic‐related factors in its prevalence. The prevalence of neonatal sepsis in this study was 69.6%, which indicates a high prevalence of sepsis among admitted neonates in a hospital and aligns with findings from earlier studies in Bangladesh [12]. Similar high prevalence rates have been reported in NICU‐based studies from other LMICs [5], where the prevalence ranges from approximately 45% to 75.4%, reflecting variations in study design, diagnostic criteria, and healthcare settings. We observed in this study that the prevalence of EONS and LONS was 60.9% and 39.1%, respectively. A previous study at Chittagong Medical College Hospital in 2011, Bangladesh, reported almost similar results for EONS (65.38%) and LONS (34.62%) [15]. The high prevalence of EONS in this study is consistent with regional data indicating that maternal‐to‐neonatal transmission of infections during delivery significantly contributes to sepsis within the first 72 h of life [9, 16]. The relatively high overall prevalence observed in this study is likely attributable to the NICU‐based sampling, where critically ill and high‐risk neonates are overrepresented. Moreover, the inclusion of both culture‐positive and clinically diagnosed sepsis cases may have contributed to a higher estimated prevalence compared to population‐based studies. Although healthcare facilities have improved in Bangladesh compared to the last decade, the occurrence of neonatal sepsis in Bangladesh is still high. The findings of this study also align with previous studies conducted in other LMICs, where newborn sepsis is a predominant cause of neonatal mortality and morbidity [17, 18].

Several issues, such as neonate's birth weight, maternal educational status, family income, and place of delivery, were identified as statistically significant risk factors for newborn sepsis in this study. Among these, neonates with low birth weight (1.5–2.4 kg) exhibited the highest risk of sepsis compared to those with extremely low birth weight (1.0–1.4 kg) and normal birth weight ( ≥ 2.5 kg) neonates. It is widely acknowledged that neonates with normal birth weight are likely to have stronger immune responses and better physiological resilience, enabling them to resist infections more effectively. On the other hand, neonates with extremely low birth weight are critically vulnerable immediately after birth and are often transferred to the NICU under strict sterile conditions for intensive care and monitoring. Consequently, their prompt admission and close medical supervision may reduce their susceptibility to community‐ or environment‐acquired infections, despite their physiological immaturity. This study demonstrated that a significant proportion of neonates with extremely low birth weight (19 out of 24) were delivered in hospitals, while most neonates with low birth weight (40 out of 53) were born at home (Table S1), indicating their vulnerability to infections due to still underdeveloped immune systems and other comorbidities [1]. The immaturity of immune defenses, poor thermoregulation, and feeding difficulties common in these neonates likely contribute to an increased risk of infection. Maternal educational status showed a strong association with newborn sepsis, with the highest prevalence observed in neonates born to mothers who had only primary or no formal education. This could indicate a lack of awareness regarding proper antenatal care, hygiene practices during childbirth, and early detection of newborn danger indicators. Similar findings have been reported in a recent study from Bangladesh, where the mothers' educational level below secondary level has been identified as a significant predictor of neonatal infections due to limited health awareness and delayed care‐seeking behavior [12]. Mothers with higher levels of education are more likely to seek timely medical assistance and follow safe perinatal practices, lowering their newborns' risk of sepsis. Similarly, family income was found to be a significant key social determinant of newborn sepsis in Bangladesh. Neonates born to mothers from low‐income families exhibited a higher risk of infection among neonates, highlighting the role of socioeconomic status in access to quality maternal and neonatal healthcare [19]. We observed in this study that the place of delivery played a critical role; neonates born at home were significantly more prone to sepsis than those who were born in a hospital or clinic, most likely due to unhygienic conditions, a lack of skilled delivery attendants, and delayed recognition of neonatal infections [20]. This finding is consistent with recent regional studies showing that home deliveries, particularly in resource‐limited settings, are associated with higher risks of neonatal infections due to suboptimal infection prevention practices and limited access to skilled birth attendants. This study observed that low income, home delivery, and maternal education are all highly interconnected and significantly associated with neonatal sepsis. We found that families with low income had a higher rate of home deliveries (49 out of 88) than those with high‐income families (11 out of 37) (Table S1), and mothers with limited education were mainly from low‐income families (Table S1). These factors form a cycle where poverty restricts education and access to safe delivery, increasing newborn sepsis. These findings highlight the importance of targeted interventions to improve maternal education, economic conditions, and access to institutional deliveries in resource‐limited settings.

Gestational age was not found to be significantly associated with neonatal sepsis in this study, contrasting with earlier findings linking preterm birth to higher sepsis risk due to immature immune and physiological systems [21]. However, the study explored a higher prevalence of sepsis in term neonates compared to preterm neonates; among them, a higher proportion of term neonates were born at home (Table S1), where hygienic conditions and skilled birth attendance are often lacking. Additionally, a majority of term neonates (65 out of 90) came from lower‐income families, whereas only 23 (out of 35) preterm neonates came from the same income group (Table S1). Similarly, we also found that the neonate's sex, maternal age, mother's occupation, and mode of delivery were not strongly associated with neonatal sepsis in our findings. These findings are in contrast to the outcomes of some previous studies [22, 23]. This discrepancy might be due to the relatively small sample size or the specific characteristics of the population that was considered for the study. However, the higher sepsis rate among neonates born via normal delivery suggests a need for further investigation into infection control during childbirth.

This study has several limitations, even though it provides valuable insights into the prevalence and risk factors of neonatal sepsis in Bangladesh. First, the sample size of the study was relatively small, and it was also a NICU‐based study. So, there is a possibility of selection bias, since critically ill and high‐risk neonates are more likely to be admitted to tertiary care facilities, which may overestimate the prevalence of neonatal sepsis compared to community‐based settings. Second, the study was conducted in a single tertiary care hospital, which may limit the generalizability of the findings to other settings and does not reflect the whole population of the country. The single‐center nature of the study may also limit the external validity of the results, as variations in clinical practices and healthcare infrastructure across different regions of Bangladesh are not captured. Third, the reliance on clinical and laboratory criteria for diagnosing sepsis, rather than microbiological confirmation in all cases, may have led to misclassification bias. Specifically, the inclusion of both culture‐confirmed and clinically diagnosed (culture‐negative) sepsis cases may introduce diagnostic variability and potentially inflate the estimated prevalence. While clinical criteria are widely used in resource‐limited settings, using blood culture results or molecular diagnostics may improve sepsis diagnosis accuracy. In addition, some important maternal clinical variables, such as Group B Streptococcus (GBS) colonization status, intrapartum antibiotic prophylaxis (IAP), and chorioamnionitis, were not consistently available in the hospital records and therefore could not be included in the analysis. The absence of these variables may limit the comprehensive evaluation of maternal risk factors associated with neonatal sepsis. Future studies should consider a multicenter design with larger sample sizes and longitudinal follow‐up to better understand the risk factors and outcomes of neonatal sepsis in Bangladesh.

## 5 Conclusion

This study provides valuable insights into the prevalence and associated risk factors of neonatal sepsis among the admitted neonates in the NICU of Bangladesh. The high prevalence of neonatal sepsis underscores the urgent need for targeted interventions, including improved maternal education, access to institutional deliveries, and enhanced neonatal care. Future research utilizing larger, multi‐center designs is recommended to further explore regional variations in neonatal sepsis prevalence and related risk factors.

## Author Contributions

**Fahmida Akter:** methodology, data curation, formal analysis, investigation, and software. **Halima Khatun Munni:** methodology, data curation, and formal analysis. **Md Abdur Rahman Ripon:** methodology, investigation, data curation, writing – review and editing. **Sujan Banik:** methodology, formal analysis, software, visualization, and writing – original draft. **Mohammad Salim Hossain:** conceptualization, writing – review and editing, supervision, methodology, visualization, and project administration.

## Funding

The authors have nothing to report.

## Conflicts of Interest

Sujan Banik is on the Editorial Board of the Health Science Reports and a corresponding author of this article. He was not involved in any of the editorial decisions that led to this paper being accepted for publication in this journal. The other authors declare no conflicts of interest.

## Transparency Statement

The corresponding author, Sujan Banik, confirms that this manuscript is an honest, accurate, and transparent account of the study being reported; that no important aspects of the study have been omitted; and that any discrepancies from the study as planned (and, if relevant, registered) have been explained.

## Supporting information

Supporting File

## Data Availability

The data that support the findings of this study are available from the corresponding author upon reasonable request.
