# Supplementary material for: Prevalence and Associated Risk Factors of Neonatal Sepsis in a Neonatal Intensive Care Unit of a Tertiary Hospital in Bangladesh: A Cross‐Sectional Study
Source: Health Sci Rep. 2026 Jun 10;9(6):e72661. doi: 10.1002/hsr2.72661 (PMC13253977; doi:10.1002/hsr2.72661)
Supplement: Supplementary file 1 — Supporting File [file HSR2-9-e72661-s001.docx]

**Supporting Information**

**Prevalence and associated risk factors of neonatal sepsis in a neonatal intensive care unit of a tertiary hospital in Bangladesh: A cross-sectional study**

Fahmida Akter, Halima Khatun Munni, Md. Abdur Rahman Ripon, Sujan Banik*, Mohammad Salim Hossain*

*Department of Pharmacy, Noakhali Science and Technology University, Noakhali 3814, Bangladesh*

^*^**Corresponding Author**

Sujan Banik, Ph.D.

Associate Professor, Department of Pharmacy

Noakhali Science and Technology University

Noakhali 3814, Bangladesh

Tel: +8801601446918

Email: [sbanik@nstu.edu.bd](mailto:sbanik@nstu.edu.bd)

Mohammad Salim Hossain, Ph.D.

Professor, Department of Pharmacy

Noakhali Science and Technology University

Noakhali 3814, Bangladesh

Tel: +8801711200410

Email: [pharmasalim@nstu.edu.bd](mailto:pharmasalim@nstu.edu.bd)

**Table S1: Cross-tabulation of neonatal and maternal characteristics among study participants**

| Variable | Category | Home | Hospital |
| --- | --- | --- | --- |
| Birth weight (kg) | | | |
|  | 1.0–1.4 | 5 | 19 |
|  | 1.5–2.4 | 40 | 13 |
|  | ≥2.5 | 15 | 33 |
| Monthly family income | | | |
|  | Low | 49 | 39 |
|  | High | 11 | 26 |
| Gestational age | | | |
|  | Preterm | 10 | 25 |
|  | Term | 50 | 40 |
| Socioeconomic characteristics | | | |
| Variable | **Category** | **Low income** | **High income** |
| Maternal education level | | | |
|  | Illiterate | 10 | 1 |
|  | Primary | 48 | 10 |
|  | Secondary | 29 | 20 |
|  | Above secondary | 1 | 6 |
| Gestational age | | | |
|  | Preterm | 23 | 12 |
|  | Term | 65 | 25 |
